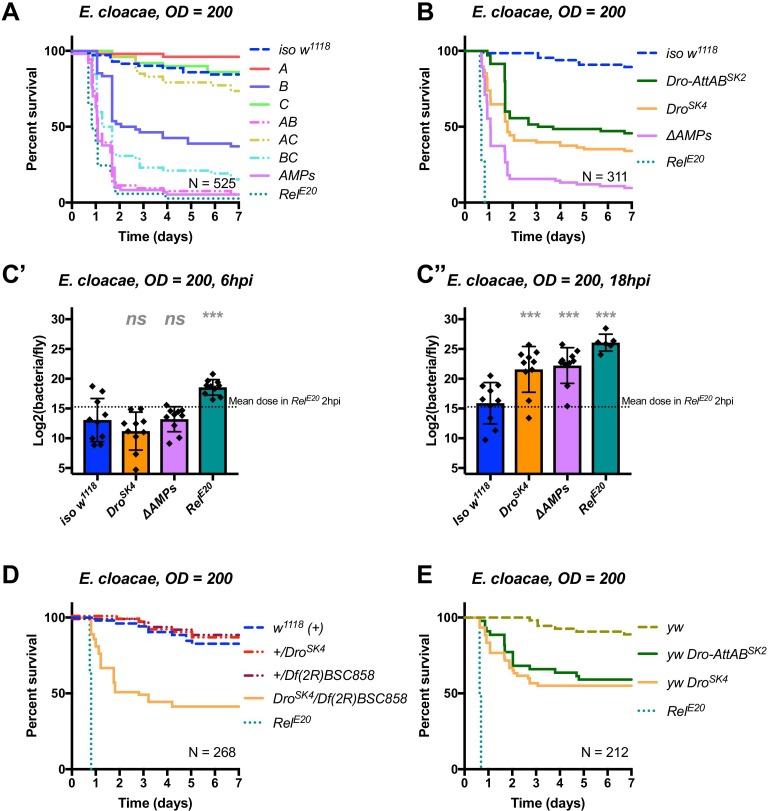# Correction: Synergy and remarkable specificity of antimicrobial peptides in vivo using a systematic knockout approach

**DOI:** 10.7554/eLife.48778

**Published:** 2019-05-28

**Authors:** Mark Austin Hanson, Anna Dostálová, Camilla Ceroni, Mickael Poidevin, Shu Kondo, Bruno Lemaître

Hanson MA, Dostálová A, Ceroni C, Poidevin M, Kondo S, Lemaitre B. 2019. Synergy and remarkable specificity of antimicrobial peptides in vivo using a systematic knockout approach. *eLife* **8**:e44341 . doi: 10.7554/eLife.44341.

Published February 26, 2019

We have recently realized that the fly stocks carrying the *Cec^SK6^* deletion (removing the genes CecA1, Cec A2, CecB and CecC) used in our recent manuscript (DOI: 10.7554/eLife.44341) carry an extra-copy of the wild-type Cecropin locus distal to the deletion. We suspect a complex recombination event that brought back the Cecropin locus nearby the deletion. Thus, flies carrying the *Cec^SK6^* deletion were in fact also carrying a wild-type locus for Cecropins. We are analyzing the exact nature of this unfortunate recombination event and will reassess the function of Cecropin in host defense. This finding does not affect the main conclusions of our study except that we do not make any conclusions regarding Cecropin and host defence. Compound mutants that we have used including the ΔAMP flies were wild-type for Cecropin. The contribution of Cecropins to the overall antimicrobial peptide defense, as well as eventual synergy with other peptides or any specific roles for this family of peptides will require us to re-generate novel *Cecropin* deficient fly lines. We apologize for only discovering this after publication. We intend to perform a careful exploration of the Cecropins in the future. We would like to express our sincere gratitude to Steven Wasserman and Scott Lindsay who first alerted us to this matter.

The article has been corrected accordingly.

A summary of the corrections to the manuscript is provided in the list below, and both the original text and corrected text are included further below for the specifics of each correction:

Summary of changes:

- References to the *Cec^SK6^* mutation as a Cecropin mutant have been removed from the manuscript, including from the following figures:
  - Figure 1A
  - Figure 1 supplement
  - Figure 4 supplement A
- Figure 1 is clarified to note we did not delete the Cecropin cluster, and we note that there are many short peptides induced by the *D. melanogaster* immune response awaiting description, including potential AMPs
- Clarified discussion of the interaction between the Toll-responsive peptides Drs and Mtk (Group C) and Defensin (Group A) observed in both Figure 3A and Figure 4A
- Figure 4 - figure supplement 1 clarifies that the aberrant Cecropin locus present in Group A flies, but not in *Def^SK3^* individual mutants, does not significantly impact susceptibility to *P. burhodogranarea*.
- New emphasis on the role of Defensin is included, as Defensin mutation alone is already susceptible to *P. burhodogranariea* (Figure 4), and *Def^SK3^* exacerbates the susceptibility of combined *Dro, Att,* and *Dpt* mutants (Group B) in Figure 6A.

Specific changes

**Abstract:**

Revised text:

Antimicrobial peptides (AMPs) are host-encoded antibiotics that combat invading microorganisms. These short, cationic peptides have been implicated in many biological processes, primarily involving innate immunity. In vitro studies have shown AMPs kill bacteria and fungi at physiological concentrations, but little validation has been done in vivo. We utilized CRISPR gene editing to delete most known immune-inducible AMPs of *Drosophila,* namely: 4 Attacins, 2 Diptericins, Drosocin, Drosomycin, Metchnikowin and Defensin. Using individual and multiple knockouts, including flies lacking these ten AMP genes, we characterize the in vivo function of individual and groups of AMPs against diverse bacterial and fungal pathogens. We found that *Drosophila* AMPs act primarily against Gram-negative bacteria and fungi, contributing either additively or synergistically. We also describe remarkable specificity wherein certain AMPs contribute the bulk of microbicidal activity against specific pathogens, providing functional demonstrations of highly specific AMP-pathogen interactions in an in vivo setting.

Original text:

Antimicrobial peptides (AMPs) are host-encoded antibiotics that combat invading microorganisms. These short, cationic peptides have been implicated in many biological processes, primarily involving innate immunity. In vitro studies have shown AMPs kill bacteria and fungi at physiological concentrations, but little validation has been done in vivo. We utilized CRISPR gene editing to delete all known immune-inducible AMPs of *Drosophila,* namely: 4 Attacins, 4 Cecropins, 2 Diptericins, Drosocin, Drosomycin, Metchnikowin and Defensin. Using individual and multiple knockouts, including flies lacking all 14 AMP genes, we characterize the in vivo function of individual and groups of AMPs against diverse bacterial and fungal pathogens. We found that *Drosophila* AMPs act primarily against Gram-negative bacteria and fungi, contributing either additively or synergistically. We also describe remarkable specificity wherein certain AMPs contribute the bulk of microbicidal activity against specific pathogens, providing functional demonstrations of highly specific AMP-pathogen interactions in an in vivo setting.

**eLife digest:**

Revised text:

In the experiments, ten antimicrobial peptide genes known from fruit flies were removed, and the flies were then infected with a variety of bacteria and fungi.

Original text:

In the experiments, all 14 antimicrobial peptide genes known from fruit flies were removed, and the flies were then infected with a variety of bacteria and fungi.

**Changes to text in the main article:**

**Introduction:**

Revised text:

In this paper, we took advantage of recent gene editing technologies to delete most of the known immune inducible AMP genes of *Drosophila*.

Original text:

In this paper, we took advantage of recent gene editing technologies to delete each of the known immune inducible AMP genes of *Drosophila*.

**Results:**

Revised text:

We generated null mutants for 10 of the 14 known *Drosophila* antimicrobial peptide genes that are induced upon systemic infection. These include five single gene mutations affecting *Defensin (Def^SK3^*), *Attacin C* (A*ttC^Mi^*), *Metchnikowin (Mtk^R1^*), *Attacin D (AttD^SK1^*) and *Drosomycin (Drs^R1^*), respectively, and two small deletions removing both *Diptericins DptA* and *DptB (Dpt^SK1^*), or the gene cluster containing *Drosocin,* and *Attacins AttA* and *AttB (Dro-AttAB^SK2^*). The function of Cecropins were not assessed in this manuscript.

Original text:

We generated null mutants for 14 *Drosophila* antimicrobial peptide genes that are induced upon systemic infection. These include five single gene mutations affecting *Defensin (Def^SK3^*), *Attacin C* (A*ttC^Mi^*), *Metchnikowin (Mtk^R1^*), *Attacin D(AttD^SK1^*) and *Drosomycin (Drs^R1^*), respectively, and three small deletions removing both *Diptericins DptA* and *DptB (Dpt^SK1^*), the four *Cecropins CecA1, CecA2, CecB, and CecC (Cec^SK6^*) and the gene cluster containing *Drosocin,* and *Attacins AttA* and *AttB (Dro-AttAB^SK2^*).

Revised text:

Then, we recombined these seven independent mutations into a background lacking these 10 inducible AMPs referred to as ‘*ΔAMPs*.’

Original text:

Then, we recombined these eight independent mutations into a background lacking these 14 inducible AMPs referred to as ‘*ΔAMPs*.

Revised text:

Given seven independent AMP mutations, over 100 combinations of mutants are possible, making a systematic analysis of AMP interactions a logistical nightmare. Therefore, we designed an approach that would allow us to characterize their contributions to defence by deleting groups of AMPs. To this end, we generated three groups of combined mutants: A) flies lacking *Defensin* (Group A); *Defensin* is regulated by Imd signalling but is primarily active against Gram-positive bacteria in vitro (Imler and Bulet, 2005). B) Flies lacking three antibacterial and structurally related AMP families: the Proline-rich *Drosocin* and the Proline- and Glycine-rich *Diptericins* and *Attacins* (Group B, regulated by the Imd pathway). C) Flies lacking the two antifungal peptide genes *Metchnikowin* and *Drosomycin* (Group C, mostly regulated by the Toll pathway).

Original text:

Given eight independent AMP mutations, over 250 combinations of mutants are possible, making a systematic analysis of AMP interactions a logistical nightmare. Therefore, we designed an approach that would allow us to characterize their contributions to defence by deleting groups of AMPs. To this end, we generated three groups of combined mutants: flies lacking the primarily antibacterial *Defensin* and *Cecropins* (Group A, mostly regulated by the Imd pathway), flies lacking the antibacterial Proline-rich *Drosocin*, and the antibacterial Glycine-rich *Diptericins* and *Attacins* (Group B, regulated by the Imd pathway), and flies lacking the two antifungal peptide genes *Metchnikowin* and *Drosomycin* (Group C, mostly regulated by the Toll pathway).

Revised text:

Curiously, AC-deficient flies that also lack *Defensin* survived better than Group C-deficient flies (Log-Rank p=0.014).

Original text:

Curiously, AC-deficient flies that also lack *Cecropins* and *Defensin* survived better than Group-C-deficient flies (Log-Rank p=0.014).

Revised text:

Flies lacking *Defensin* (Group A) showed an intermediate susceptibility, but behave as wild-type in the additional absence of Toll Group C peptides (Group AC). Thus, we again observed a better survival rate with the co-occurring loss of Group A and C peptides (see possible explanation above). In this case, Group A flies were susceptible while AC flies were not.

Original text:

Flies lacking *Defensin* and the four *Cecropins* (Group A) showed an intermediate susceptibility, but behave as wild-type in the additional absence of Toll Group C peptides (Group AC). Thus, we again observed a better survival rate with the co-occurring loss of Group A and C peptides (see possible explanation above). In this case, Group A flies were susceptible while AC flies were not. Flies individually lacking *Defensin* or the four *Cecropins* were weakly susceptible to *P. burhodogranariea* (p=0.022 and p=0.040, respectively); however, the interaction term between *Defensin and* the *Cecropins* was not significant (Def^SK3^*Cec^SK6^, HR = −0.28, p=0.382), indicating the susceptibility of Group A flies arises from additive loss of resistance (Figure 4—figure supplement 1A).

Revised text:

Meanwhile, Group AB flies additionally lacking *Defensin* reached *ΔAMPs* levels of susceptibility, while Group A and Group C flies resisted as wild-type (Figure 6A). The high susceptibility of Group AB flies results from a synergistic statistical interaction amongst Group A (Defensin) and Group B peptides in defence against E. cloacae (A*B, HR =+2.55, p=0.003).

Original text:

Meanwhile, Group AB flies reached *ΔAMPs* levels of susceptibility, while Group A and Group C flies resisted as wild-type (Figure 6A). The high susceptibility of Group AB flies results from a synergistic statistical interaction amongst Group A and Group B peptides in defence against E. cloacae (A*B, HR =+2.55, p=0.003).

**Discussion:**

Revised text:

With seven distinct mutations, we were able to generate a fly line lacking 10 AMPs that are known to be strongly induced during the systemic immune response.

Original text:

With eight distinct mutations, we were able to generate a fly line lacking 14 AMPs that are known to be strongly induced during the systemic immune response.

Revised text:

We found activity of Diptericins against *P. rettgeri*, Drosocin against *E. cloacae*, Drosomycin and Metchnikowin against *C. albicans*, and Defensin against *P. burhodogranariea*.

Original text:

We found activity of Diptericins against *P. rettgeri*, Drosocin against *E. cloacae*, Drosomycin and Metchnikowin against *C. albicans*, and Defensin and Cecropin against *P. burhodogranariea* (Figure 4—figure supplement 1A).

Revised text:

Consistent with this, *ΔAMPs* flies are almost as susceptible as Imd-deficient mutants to most Gram-negative bacteria. In contrast, flies lacking AMPs were only slightly more susceptible to Gram-positive bacteria and fungal infections compared to wild-type flies, and this susceptibility rarely approached the susceptibility of *Bomanin* mutants. It is possible that additional loss of *Cecropins* would further increase the sensitivity of *ΔAMPs* flies to bacteria or fungi.

Original text:

Consistent with this, *ΔAMPs* flies are almost as susceptible as Imd-deficient mutants to most Gram-negative bacteria. In contrast, flies lacking AMPs were only slightly more susceptible to Gram-positive bacteria and fungal infections compared to wild-type flies, and this susceptibility rarely approached the susceptibility of *Bomanin* mutants.

Revised text:

*Drosophila* Drosocin is highly similar to Abaecin and the related peptide Apidecin, including O-glycosylation of a critical threonine residue (Imler and Bulet, 2005; Hanson et al., 2016), and thus likely acts in a similar fashion.

Original text:

*Drosophila* Drosocin is highly similar to Abaecin, including O-glycosylation of a critical threonine residue (Imler and Bulet, 2005; Hanson et al., 2016), and thus likely acts in a similar fashion.

Revised text:

Astoundingly, flies mutant for the other inducible AMPs resisted *P. rettgeri* infection as wild-type, while only *Diptericin* mutants succumbed to infection.

Original text:

Astoundingly, flies mutant for all other inducible AMPs resisted *P. rettgeri* infection as wild-type, while only *Diptericin* mutants succumbed to infection.

**Materials and Methods:**

Revised text:

In brief, flies deficient for *Drosocin, Attacin A,*and *Attacin B (Dro-AttAB^SK2^),* and *Diptericin A* and *Diptericin B (Dpt^SK1^)* were produced by gene region deletion specific to those AMPs without affecting other genes.

Original text:

In brief, flies deficient for *Drosocin, Attacin A,*and *Attacin B (Dro-AttAB^SK2^), Diptericin A* and *Diptericin B (Dpt^SK1^)*, and *Cecropins CecA1, CecA2, CecB, CecC (Cec^SK6^)* were all produced by gene region deletion specific to those AMPs without affecting other genes.

Revised text:

Mutations were isogenized for a minimum of seven generations into the *iso w^1118^* background prior to subsequent recombination. It should be noted that Group A flies were initially thought to be a double mutant for both *Defensin* and the *Cecropin* cluster, resulting from a combination of *Def^SK3^* and a CRISPR-induced *Cecropin* deletion (called *Cec^SK6^*). It was subsequently shown that *Cec^SK6^* is a complex aberration at the *Cecropin* locus that retains a wild-type copy of the *Cecropin* cluster. This re-arranged *Cecropin* locus does not contribute significantly to the susceptibility of Group A flies, as Group A was not different from *Def^SK3^* alone (Log-Rank p=0.818; Figure 4—figure supplement 1A). Thus, group A flies were considered as single *Def^SK3^*mutants.

Original text:

Mutations were isogenized for a minimum of seven generations into the *iso w^1118^* background prior to subsequent recombination.

**Changes to text in the figure legends:**

**Figure 4**

Revised text:

Loss of the Group A peptide Defensin also resulted in strong susceptibility (p<0.001) (and see Figure 4—figure supplement 1).

Original text:

Loss of Group A peptides also resulted in strong susceptibility (p<0.001) due to additive effects of Defensin and Cecropins (Figure 4—figure supplement 4).

**Figure 4 – figure supplement 1**

Revised text:

(A) Group A flies (here labelled *Def^SK3^; Cec^SK6^*) have an abberant Cecropin locus (*Cec^SK6^*), but this contributes little to survival compared to *Def^SK3^* mutants (p=0.818). *Def^SK3^* flies are susceptible to *P. burhodogranariea* (Log-Rank p=0.022) (B) Upon infection with the Gram-negative *Ecc15*, Group B peptides (Drosocin, Attacins and Diptericins) explain the bulk of mortality, but additional loss of other peptides in *ΔAMPs* flies leads to increased mortality (Log-Rank p=0.013).

Original text:

(A) Dissection of the susceptibility of Group A flies lacking *Defensin* and *Cecropins* reveals that combined mutants have an additive loss of resistance (Def*Cec, HR =+0.36, p=0.342). (B) Upon infection with the Gram-negative *Ecc15*, Group B peptides (Drosocin, Attacins and Diptericins) explain the bulk of mortality, but additional loss of other peptides in *ΔAMPs* flies leads to increased mortality (Log-Rank p=0.013).

**Modified figures:**

**Figure 1**: Removed *Cec^SK6^* from Figure 1A

The corrected Figure 1 is shown here:

**Figure 1:**
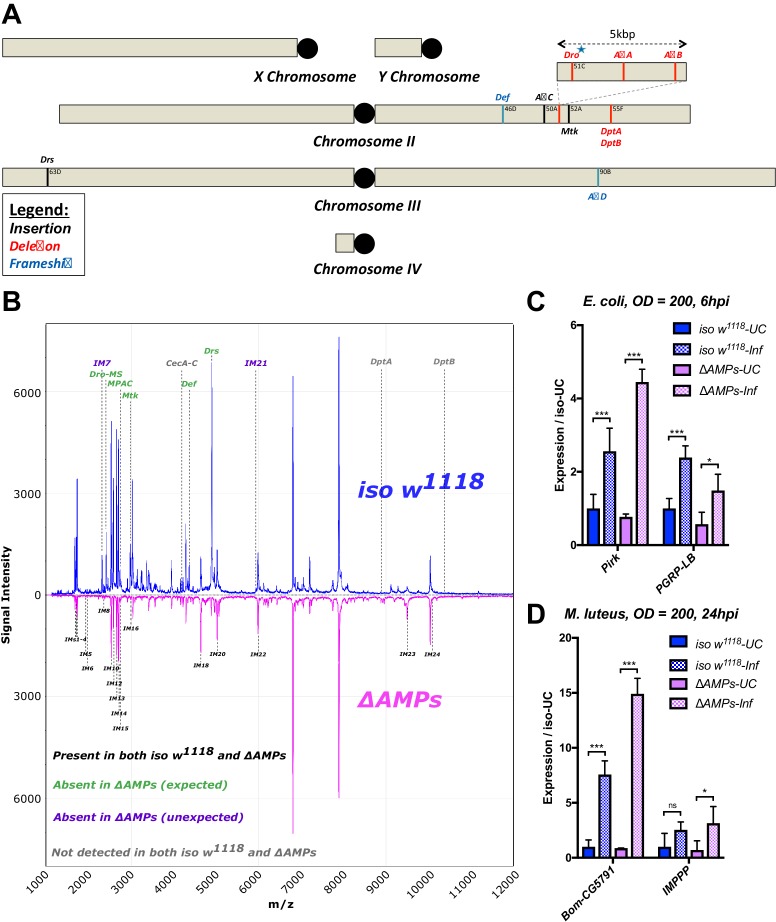


The originally published Figure 1 is shown here:

**Figure 2:**
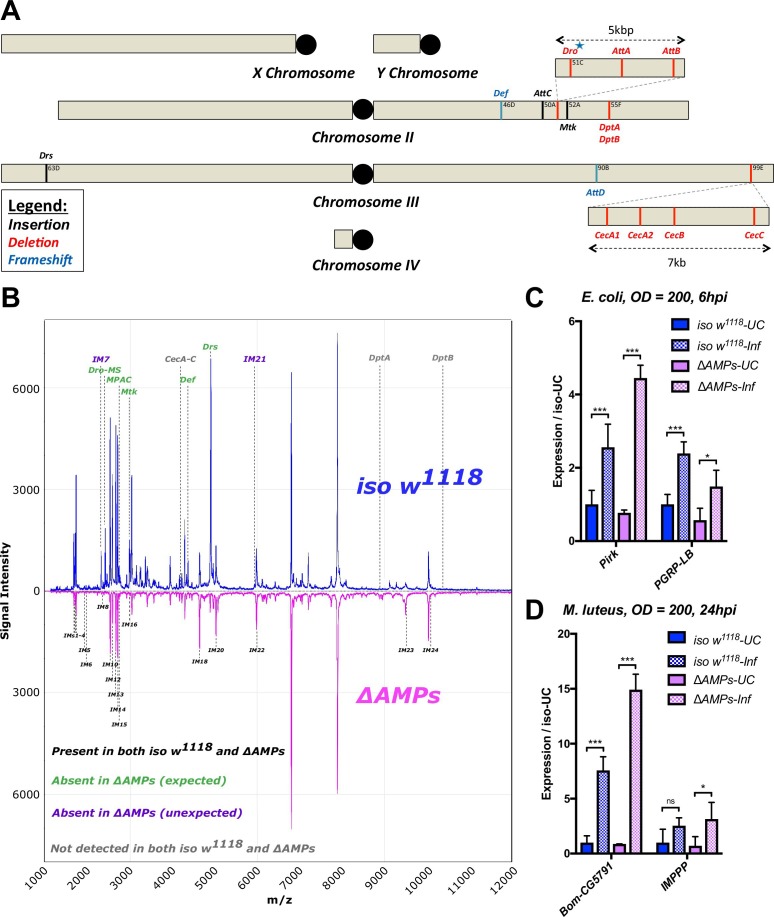


**Figure 1 - figure supplement 1**: Removed *Cec^SK6^* from figure

The corrected Figure 1 - figure supplement 1 is shown here:

**Figure 3:**
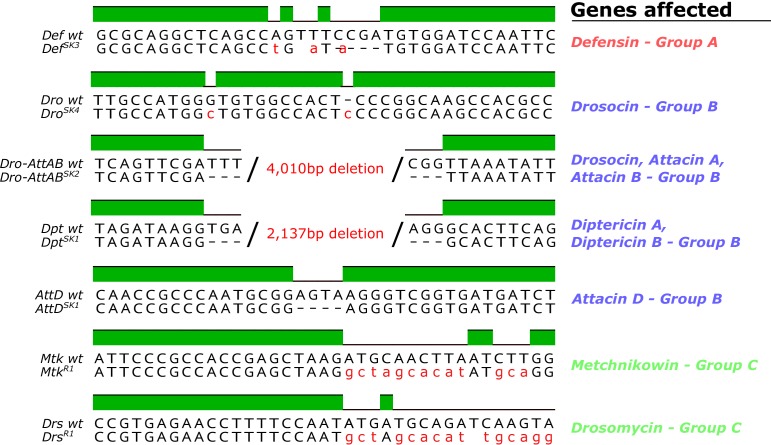


The originally published Figure 1 - figure supplement 1 is shown here:

**Figure 4:**
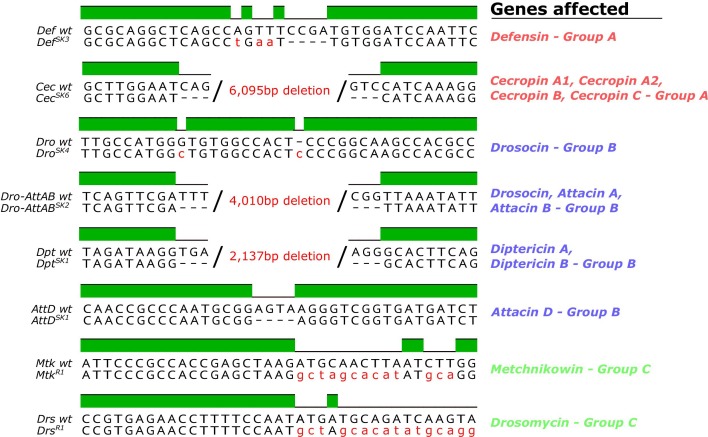


**Figure 4 – figure supplement 1A**: Removed *Cec^SK6^* from figure, modified labels to reflect mutation status better.

The corrected Figure 4 – figure supplement 1 is shown here:

**Figure 5:**
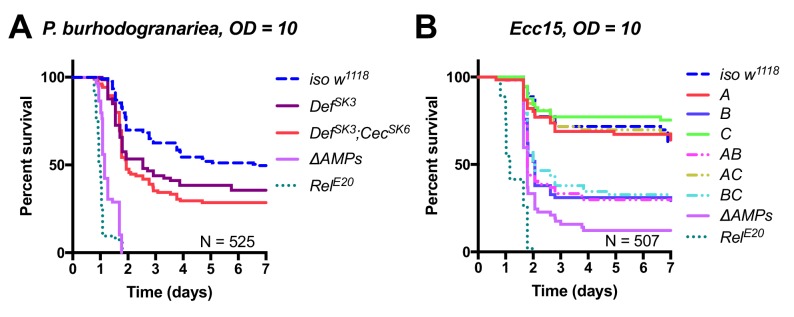


The originally published Figure 4 – figure supplement 1 is shown here:

**Figure 6:**
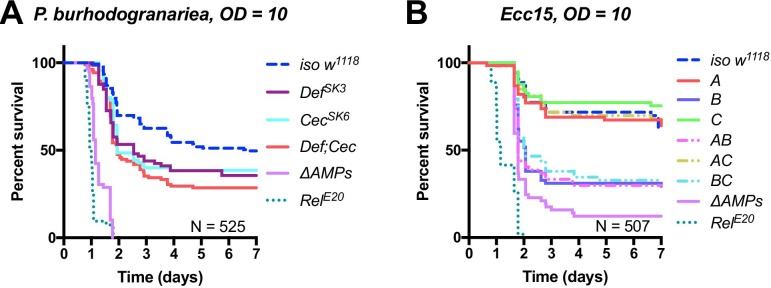


**Figure 6**: Corrected typo in Figure 6A labelling *ΔAMPs*

The corrected Figure 6 is shown here:

**Figure 7:**
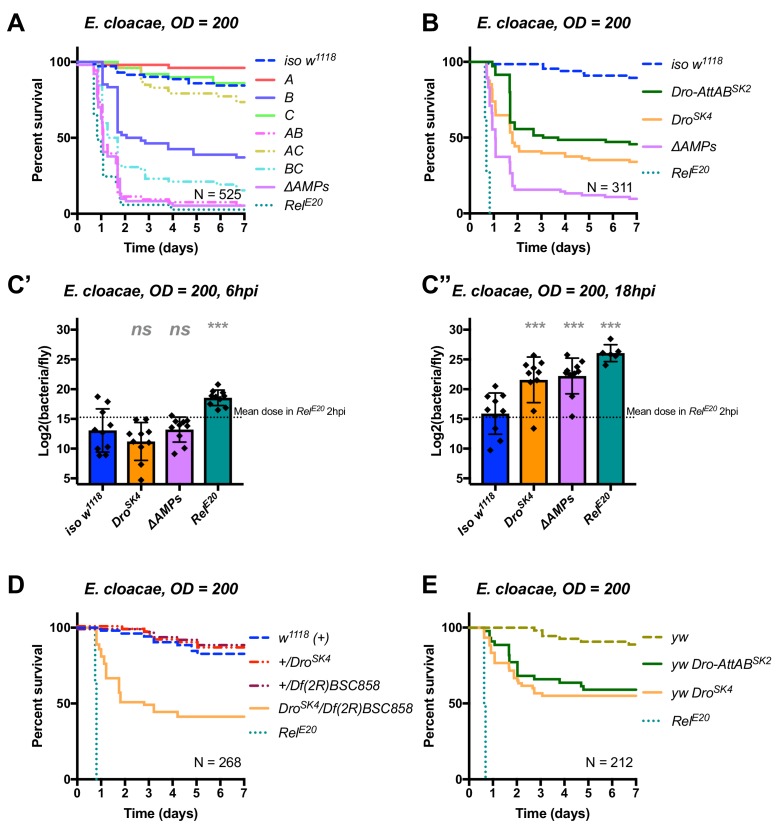


The originally published Figure 6 is shown here:

**Figure 8:**